# Puerperal Eclampsia

**Published:** 1886-03

**Authors:** J. W. Carhart

**Affiliations:** Lampasas, Texas


### PUERPERAL ECLAMPSIA.

*By J. W. Carhart, M. D., of Lampasas, Texas.*

For Daniel's Texas Medical Journal.

SHOULD an apology seem to be needed for the presentation of a subject as familiar to the medical profession as puerperpal eclampsia, we have only to refer to the statement of Leishman, in his work on Midwifery, p. 660, viz.: "The maternal and fœtal mortality arising from this disease are (is a) subject(s) of great and obvious interest, since about thirty per cent of mothers have hitherto succumbed to its effects, direct or indirect."

Since the ablest writers on the subject differ widely in their views as to the pathology of puerperal eclampsia, we shall not attempt to enlighten our brethren in regard to it; but a few suggestions as to the ætiology of the disease and our duty as physicians, may not be out of place.

It is not yet a fully established fact that albuminuria is the cause of eclampsia, but it is certain that albuminuria and puerperal eclampsia seem to be mutually dependent; that is to say, where we find puerperal eclampsia we invariably have albuminurea; and where we have a certain per cent of albumen in the urine of pregnant women, we are just as certain to have eclampsia.

Braum claims that albumen appears in the urine as the result of the inflammatory affection of the kidneys, known as Bright's disease; and that, as a result of this, the blood is poisoned with excrementitious matter from the urine, especially with urea, and that puerperal eclampsia results from uræmic poisoning. Others, however, have shown that the presence of urea in the blood, in considerable quantities, does not give rise to eclampsia.

Whatever, then, may be the true pathology and the real ætiology of puerperal eclampsia, the interdependence of the two conditions, albuminurea and this affection, it is a fact of such vast importance that we are justified in emphasizing it, and in inferring certain duties which we as physicians are sacredly bound to observe.

According to the statistics of England and the Continent, there is one case of eclampsia in every 350 labors. At first sight it would seem a little difficult to harmonize these figures with the statement of Leishman, that thirty per cent of mothers succumb to this disease, directly or indirectly. Assuming, however, that those perishing of puerperal eclampsia bear, on an average, $11\frac{2}{3}$ children each, the figures would harmonize.

The relative frequency of the disease may be divided into three periods, in the following order: Pregnancy, labor, after delivery. The disease is thought to be less dangerous after delivery than during the other two epochs.

It frequently happens that a physician is called after labor has actually commenced, and then sees the woman for the first time. It not infrequently occurs, in the practice of midwives, that the woman is actually in convulsions before a physician is summoned, when he must carry the case to successful issue, and gain some applause, or else the woman dies on his hands, and he suffers in his reputation, to some extent.

In many cases a physician is engaged a month or two before expected confinement; but he is given to understand that his services will not be needed until the event occurs, as " she is in perfect health."

If a request be made for a specimen of urine, it is either refused, or the request, through carelessness, is not complied with. Thus the large number of mothers and children who perish in the hands of ignorant midwives is added to by the ignorance and obstinacy of expectant mothers and their friends and the carelessness of physicians.

Whilst it is true that a complete cure of albuminuria could not be effected, though the physician was summoned at an early stage of pregnancy, it could in many cases be so modified and alleviated as to insure the comparatively safe passage of the mother through the ordeal of confinement, and give to the physician a chance to fortify himself against unjust criticism, in any event.

The recognized treatment for albuminuria being clearly presented in all the books on midwifery, aud accessible to all, we will not consume time by a reference to it here, further than to say that we have had good results in the use of the following:

℞ Chloral hydrate..............gr. x—Sig.

Three times a day.

In connection with the following, where there was cardiac weakness or renal inadequacy:

℞ Tinct. digitalis..................ʒj

Fluid Ext. Scillæ.............℥ss

" " Conii............m 3 2

Water q. s.....................℥jj—M. Sig.

Teaspoonful twice a day.

Of course, if spasms occur, chloroform, by inhalation, to avert the attacks is the sheet anchor; and in occasional instances blood-letting can be resorted to with good results.

Pilocarpine may be used with good results when the heart's action is not too much depressed, as was done by W. C. Fisher, M. D., of Galveston, Texas, and reported in the *Texas Courier-Record of Medicine* for January, 1886, p. 189. His formula was as follows:

℞ Pilocarpine muriate.........gr. ss

Sp'ts frumenti.................℥ j

Aqua ad.... ................℥iij—M. Sig.

Teaspoonful every three hours.

On Sunday morning, January 31, 1886, at 3 o'clock, I was summoned to attend Mrs. E———, in confinement. She was white, a native of Texas, twenty-eight years of age, a primipara. I had no knowledge of her, and never saw her until I entered her room.

On making the usual examination, I noticed that there was œdema of the feet and legs, and that there were varicose veins, and that there had been a number of varicose ulcers on both legs. I expressed surprise to her lady friends in attendance that she had not had a physcian sooner. The ladies told the husband, who spoke to me about it; to whom I made the statement that she should have had a physician months before, and that, although I was apprehensive of some trouble, I hoped she would come through all right. The labor was no more severe than I expected, in view of her age and the fact of her being a primipara.

She was safely delivered of a well-developed child, without the use of instruments and without laceration. The labor was somewhat tedious, but not unusually so; and she behaved badly and suffered herself to become considerably excited. The placenta came away in about thirty minutes, without any difficulty, and the uterus contracted firmly, and every thing was in as favorable condition as could be desired.

I left the house at 10:30 a. m. At 2:30 p. m. the husband rushed into my office and said his wife was dying. I was at her bed-side in a few minutes, and found her pulse regular and strong, the patient rational, but suffering from severe headache. The ladies in attendance, a dozen or more, were very much excited, and could not give an intelligent account of what had happened; but I inferred that she had had a spasm. My fears were confirmed, for in a few moments she was seized with another attack. I applied chloroform as soon as respiration was sufficiently restored to warrant it. I remained with her most of the time until 9:30 p. m., when I succeeded in warding off the attacks. At this time the pulse was strong and regular, and there was every indication that she would recover. There were twelve women and three boys present in the room, several men outside, and a number of carriages at the door. Two other physicians had been sent for without my knowledge, and soon appeared upon the scene. One of them was of such repute that I would neither counsel with nor recognize him. He had been called at the suggestion of some of the neighbor women. The husband and immediate friends wished me to remain in charge of the case, but desired me to recognize the obnoxious practitioner. I assured them, as did also my friend, Dr. J., that she would recover, with proper treatment, and we withdrew from the case. She has not yet recovered.

The following suggestions would seem to be pertinent:

1. What, if anything, can be done to induce parties to engage physicians earlier to attend obstetrical cases, and to encourage a test of urine for albumen.

2. Are physicians justifiable, under ordinary circumstances, in refusing to attend obstetrical cases, when called at the last moment, to women they never saw before, and of whose condition of health they know nothing?

